# Glucocorticoid exposure predicts survival in female baboons

**DOI:** 10.1126/sciadv.abf6759

**Published:** 2021-04-21

**Authors:** Fernando A. Campos, Elizabeth A. Archie, Laurence R. Gesquiere, Jenny Tung, Jeanne Altmann, Susan C. Alberts

**Affiliations:** 1 Department of Anthropology, University of Texas at San Antonio, San Antonio, TX 78249-1644, USA.; 2 Department of Biology, Duke University, Durham, NC 27708, USA.; 3 Department of Biological Sciences, University of Notre Dame, Notre Dame, IN, 46556, USA.; 4 Population Research Institute, Duke University, Durham, NC 27708, USA.; 5 Department of Evolutionary Anthropology, Duke University, Durham, NC 27708, USA.; 6 Canadian Institute for Advanced Research, 661 University Ave., Suite 505, Toronto, ON M5G 1M1, Canada.; 7 Department of Ecology and Evolutionary Biology, Princeton University, Princeton, NJ 08544, USA.

## Abstract

Are differences in hypothalamic-pituitary-adrenal (HPA) axis activation across the adult life span linked to differences in survival? This question has been the subject of considerable debate. We analyze the link between survival and fecal glucocorticoid (GC) measures in a wild primate population, leveraging an unusually extensive longitudinal dataset of 14,173 GC measurements from 242 adult female baboons over 1634 female years. We document a powerful link between GCs and survival: Females with relatively high current GCs or high lifelong cumulative GCs face an elevated risk of death. A hypothetical female who maintained GCs in the top 90% for her age across adulthood would be expected to lose 5.4 years of life relative to a female who maintained GCs in the bottom 10% for her age. Hence, differences among individuals in HPA axis activity provide valuable prognostic information about disparities in life span.

## Teaser

In wild female baboons, high fecal glucocorticoid concentrations measured repeatedly across adulthood predict shorter life spans.

## INTRODUCTION

The idea that chronic activation of the hypothalamic-pituitary-adrenal (HPA) axis [a component of the “stress response” (*1*)] is bad for individual health and survival has become ingrained in the popular understanding of stress (*2*, *3*). Yet, whether individual differences in HPA axis activation contribute to survival disparities in natural animal populations has been the subject of considerable scientific debate (*4*–*6*). In all vertebrates, hormonal mediators of the stress response play essential roles, enabling organisms to respond effectively to environmental and social challenges. Despite the highly conserved nature of the HPA axis, the established link between HPA axis dysfunction and stress-related disease in humans and laboratory animals supports the pathogenic potential of some types of endocrine response (*7*, *8*). The prevailing model to explain this link (hereafter the “chronic stress hypothesis”) posits that chronic activation of the HPA axis in response to frequent or sustained exposure to stressors results in glucocorticoid (GC) dysregulation and, ultimately, compromised health and survival (*9*). Evidence for the chronic stress hypothesis stems from two largely disconnected fields. First, psychologists and social epidemiologists find that chronic exposure to adverse conditions—including social isolation, low socioeconomic status, and social subordination—are associated with increased activation of the HPA axis and GC secretion (*10*). Second, endocrinologists find that chronically elevated GCs have a wide range of detrimental effects on health, including cardiovascular disease, type 2 adult-onset diabetes, and dysregulated immune function (*11*, *12*).

On the other hand, support for the chronic stress hypothesis is nearly absent when it comes to explaining survival disparities in natural populations of animals (*4*, *13*). In addition, no prospective tests in humans have yet shown a link between chronically elevated GCs and shortened life span. Recent reviews have highlighted the challenges associated with marshalling such support, especially when individuals occupy different time periods, locations, or environments (*4*, *5*, *14*). One common complication is that if environmental stressors affect both fitness outcomes and endocrine traits, then the fitness-reducing effects of environmental stressors can be erroneously misinterpreted as a direct effect of the endocrine response itself, when in fact a robust endocrine response may enhance survival relative to a weaker response (*15*). As a result, several authors have questioned whether the pathways that connect social adversity in humans with poor health or survival can be explained by the chronic stress hypothesis (*16*, *17*).

Further, prior studies have been hampered by two key limitations to testing relationships between exposure to stressors, HPA axis activity, and Darwinian fitness. First, most studies assess HPA axis activity by measuring GCs once or twice in a subject’s life, and single measures of GC are generally less closely associated with social factors than are repeated measures of GC (*18*). Second, the only study to prospectively evaluate these relationships used a chronic psychosocial stress model in laboratory-housed mice (*19*). Stressors imposed in captivity can differ considerably from those experienced by natural human or animal populations (*20*). Hence, we still do not know whether the links between experimental stress models, HPA axis activity, and mortality identified in experimental animal models can be extended to humans or other unmanipulated vertebrate populations.

Filling this gap is important not only for human health but also for ecology and evolution: After decades of measuring the causes of GC variation in wild vertebrates, ecologists have been challenged to show how such variation relates to Darwinian fitness (*4*, *5*, *13*, *14*). There is a growing view that animals in natural conditions do not experience adverse health or fitness consequences associated with chronically elevated GCs because strong selection for plasticity enables each individual to mount an optimal or near-optimal GC response to its current environmental challenges (*4*, *6*, *21*).

**Fig. 1:**
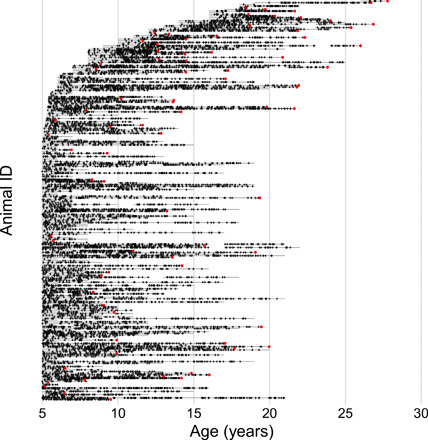
Longitudinal sampling of fGCs during the adult lives (≥age 5) of 242 female baboons included in this study. Each female study subject is represented by a horizontal line showing periods of continuous observation of that subject, and small black crosses show fGC samples for that subject (*N* = 14,173). Red dots that terminate lines indicate deaths, and lines without a terminal dot indicate censored records (that is, females who were alive at the end of the observation period). The lines are ordered by age at entry into the study. The data begin after age 5 for females who were already adults when fGC sample collection began in 1999. Small gaps in the timelines of certain females represent infrequent interruptions of data collection (e.g., during political disruptions).

Here, we link GC measures to natural life span in a longitudinal dataset consisting of 14,173 GC measurements and accompanying environmental, demographic, and behavioral data collected from 242 wild female baboons (*Papio cynocephalus*, with some admixture from a closely related species, the anubis baboon, *Papio anubis*) living in the Amboseli ecosystem in Kenya (see Materials and Methods) (*22*). This ecosystem, which is typical for yellow baboons, is characterized by a high degree of seasonality and experiences pronounced intra- and interannual climate variation (fig. S1). We combine repeated measures of GCs (Fig. 1; median = 7 measurements per female per year across 1634 female years; fig. S2) with survival outcomes and continuous, fine-grained data on environmental and life-history covariates throughout adulthood. In doing so, we demonstrate strong links between environmental factors, GC levels, and survival in a long-lived wild animal that is also an important model for human aging. We focus on adult survival because it is the most important determinant of individual fitness differences in baboons (*23*).

A key advance in this study is longitudinal sampling of fecal GCs (fGCs) throughout adult life (Fig. 1). Dense longitudinal sampling of GCs is difficult to achieve in humans, where studies that have assessed the relationship between survival and GCs or other biomarkers of “allostatic load” usually involve one measurement, with survival (and possibly a second measurement) assessed at a specific follow-up time [e.g., (*24*, *25*)]. Compared to GC measurements from plasma or blood, measurements from feces provide more integrative measures of GC concentrations over a period of several hours, are less prone to biases associated with restraint/handling and short-term fluctuations, and correlate more strongly with true baseline GC levels and with responses to exogenous hormonal challenges (*26*).

To test the link between HPA axis activation and longevity, we used joint modeling of longitudinal and time-to-event data (*27*). The joint modeling approach simultaneously estimates two submodels for two linked processes; in our case, (i) longitudinal fGC concentrations (a time-varying biomarker) and (ii) survival.

Our longitudinal submodel was a linear mixed-effects model of log fGC concentrations with a two-level hierarchical structure: repeated fGC measurements at irregular time points clustered within individuals. In our study population, fGCs vary in response to several environmental, social, and individual factors (age, reproductive state, social status, season, rainfall, maximum temperature, and group size) as well as sample storage conditions (*28*–*32*). We included these sources of variance as fixed effects in the longitudinal submodel [note that we did not model the effects of genetic ancestry, which varies in this hybrid population (*33*): Ancestry estimates were missing for 19% of female subjects, and separate analyses on the remaining subset indicate no discernable effects of ancestry on fGCs; see Materials and Methods]. We allowed log fGC to follow an individual-specific trajectory by including a fixed intercept and fixed slope of age, as well as a random intercept and random slope of age for each individual. The trajectories that result from the longitudinal submodel therefore describe the evolution of each female baboon’s GC “baseline” over adulthood, including the effects of known influences.

Our time-to-event submodel was a parametric proportional hazards model of all-cause mortality in adult female baboons, with a flexible baseline hazard that we approximated using b-splines (for details about ascertaining when deaths occur, see Materials and Methods). We included exogenous time-varying covariates that are known to influence the survival of adult female baboons, quantified in 1-year time steps aligned to individual birthdays (for details of the models and additional predictors, see Materials and Methods).

The relationship between the two submodels is captured by an association structure in which a specific aspect of the individual-specific biomarker trajectory is included as a time-varying predictor of survival. We fit joint models using three common and plausible association structures between the longitudinal and time-to-event submodels, including as predictors in the survival model the current value, current slope, or cumulative effect (area under the curve) up to the current time of the individual-specific fGC trajectory (fig. S3). The current value joint model tests whether high GCs at a given time point are linked to higher mortality. The cumulative effect joint model considers the individual’s history of GC values to test whether greater cumulative exposure to GCs is linked to higher mortality. Last, the current slope joint model tests whether a steep increase in GCs at a given time point is linked to higher mortality. We fit the models under a Bayesian framework using the R package rstanarm version 2.19.3 (*34*, *35*).

## RESULTS AND DISCUSSION

We found that individual fGC trajectories over adulthood were powerful predictors of survival in female baboons, in both the current value and cumulative effect joint models. In the current value joint model, in which we used the individual’s fGC baseline as a time-varying covariate of survival, each one unit increase in the individual’s model-estimated concentrations of log fGC (equal to 2.24 SDs) predicted a 2.83-fold increase in the expected hazard of death [log hazard posterior mean = 1.041, 90% credible interval (CI): [0.120, 1.964]] (fig. S4).

**Fig. 2:**
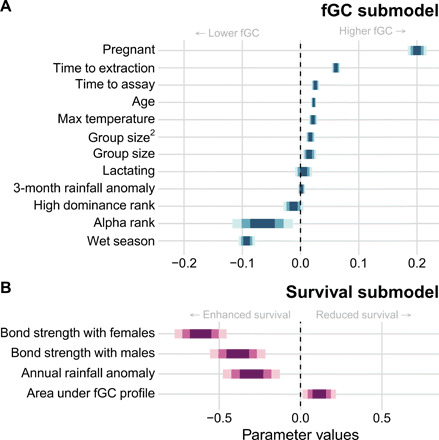
Summary of fixed parameters in the cumulative effect joint model using area under the log fGC trajectory as a time-varying predictor of survival for adult female baboons. For each estimate, the 50% (inner), 75% (middle), and 90% (outer) CIs are shown. (**A**) Posterior fixed effect parameter estimates from the linear mixed-effects submodel for ln(fGC) are listed on the *y* axis and standardized to the same scale. Estimates for levels of the categorical predictors “Pregnant” and “Lactating” are relative to the reproductive state “Cycling.” The categorical predictors “Alpha rank” and “Wet season” are relative to the condition of not being the alpha female and of the dry season, respectively; the other predictors are continuous. (**B**) Posterior fixed parameter estimates from the time-to-event submodel of the effects of time-varying covariates on the log hazard of death in adult female baboons. Social bond strength reflects the mean strength of the female’s top three social bonds with females (dyadic sociality index to females; DSI_F_) and males (DSI_M_); see Materials and Methods. “Area under fGC profile” is the association parameter that quantifies the predictive relationship between the area under the individual-specific fGC trajectory and the log hazard of death at the current time.

**Fig. 3:**
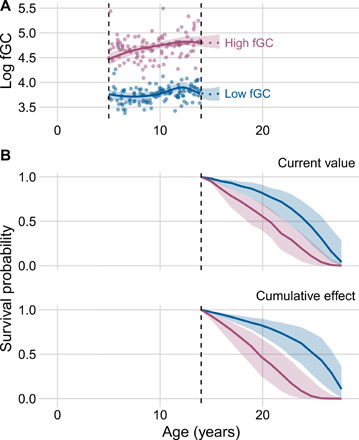
Individual-specific log fGC trajectories for simulated out-of-sample individuals and dynamic predictions of survival based on current value and cumulative effect of fGC. (**A**) Simulated log fGC profiles for two hypothetical adult female baboons that consistently maintain high (90th percentile) or low (10th percentile) fGC concentrations during each year of life until right censoring. The vertical dashed lines show age at entry “5” and age at censoring “14.” These trajectories were generated from the current value joint model of fGC and survival. (**B**) Predicted survival based on the current value (top) and cumulative effect (bottom) of fGC for the hypothetical high- and low-fGC individuals, conditional on survival to age 14, which is indicated by the vertical dashed lines. Shaded regions show 90% CIs.

The cumulative effect joint model also predicted reduced survival in individuals with high cumulative fGC exposure (log hazard posterior mean = 0.115, 90% CI: [0.012, 0.218]; Fig. 2). Although the absolute magnitude of the posterior cumulative effect size is smaller than that for the current value, their estimates cannot be directly compared because their units differ. To help interpret the cumulative effect parameter, we therefore generated individual-specific survival probabilities for hypothetical individuals with different fGC profiles [i.e., “dynamic predictions” for out-of-sample individuals (*36*)]. To do so, we simulated high and low fGC trajectories (90th and 10th percentiles for log fGC in each age class, respectively), with all continuous covariates (except age) fixed at their age-specific mean values. We also simulated individual-level random effects conditional on these data based on draws from the posterior predictive distribution. The cumulative effect model shows markedly reduced survival in the high-fGC individual compared to the low-fGC individual (Fig. 3). For example, assuming the fGC trajectories of both individuals are last observed at age 14 years, median survival probabilities reach 0.5 in the high-fGC individual at age 19.6 years and the low-fGC individual at age 25.0 years. This difference (5.4 years) is roughly equivalent to 25% of a female baboon’s life expectancy, conditional on reaching adulthood (Fig. 3). Compared to the current value model, the conditional survival probabilities generated from the cumulative effect model for simulated high- and low-fGC individuals showed a greater disparity in life expectancy (fig. S4). These findings suggest increasing costs to high fGCs across the life course, not only close to the time of death. The magnitude of this effect on survival is similar to other strong predictors of adult survival in female baboons, including being socially isolated or experiencing multiple forms of early-life adversity (*37*, *38*).

The current slope joint model tested the possibility that a strong link between GCs and survival could be explained by a spike in GC concentrations shortly before death. It is well known from clinical studies that trauma, critical illness, and other acute life-threatening conditions all lead to activation of the HPA axis (*39*). This response mobilizes stored energy to support vital organ function and is essential for survival, as critically ill patients with GC deficiencies who cannot marshal this response have poor survival outcomes (*40*). To test this possibility, we determined whether death hazard was predicted by positive slope values, suggesting a spike in the fGC trajectory before death in response to a prolonged life-threatening challenge, such as critical illness. The CIs associated with the current slope association parameter on the log hazard of death were wide and nearly centered on zero (50% CI: [−6.95, 8.17], 90% CI: [−18.60, 19.04]).

Thus, our results do not support the possibility that the elevated mortality risk associated with a higher fGC profile is a result of spikes in GC secretion associated with imminent death. Instead, our findings represent rare empirical evidence that individual differences in integrative GC measures, assessed repeatedly over time, are linked to differences in survival, after including known environmental, social, and individual sources of variance in this measure.

These findings do not demonstrate that chronically elevated GCs are maladaptive because this response may yet increase fitness relative to individuals in the same environmental circumstances who mount insufficient or inappropriate GC responses. However, the fitness costs of not experiencing an activation of the HPA axis in response to that environment would have to be large, given that the differences in expected life span for females at the extremes of the fGC distribution translate to one or more likely surviving offspring. Below, we offer two possible interpretations that represent end points along a continuum: A priority for future studies is to understand the relative contributions of these explanations to the causal pathways that link GCs with survival.

First, our findings are consistent with the long-standing hypothesis, based on human and laboratory animal studies, that negative health effects associated with repeated, sustained HPA axis activation reduce life span (*9*). In this view, some individuals show dysfunction in HPA axis regulation, leading to endocrine responses that are not optimally matched to the challenges that they face. This view explicitly posits that an “optimal” match may not be achievable for a variety of reasons, including evolutionary constraints or limits to plasticity. Individual differences in GC levels are the result of environmental, genetic, or developmental differences among individuals in their tendency to exhibit high or low levels of GCs (*41*). Although doubt remains whether animals in natural populations experience fitness costs associated with GC overproduction, converging evidence indicates that such effects are likely to exist in social primates. Socially stressed wild baboons show signs of pathological hyperactivity of the HPA axis (*42*), and in wild chimpanzees and baboons, aging is accompanied by endocrine changes that are typical of HPA axis dysfunction in humans, including up-regulation of HPA axis activity, overproduction of GCs, and blunting of GC responses (*32*, *43*). In captive macaques, experimental manipulation of social stress using controlled dominance reversals—which sometimes occur in natural populations as well—causally alters expression of genes involved in inflammation, immune function, and GC signaling, suggesting that such effects are likely to occur in nature as well (*44*, *45*).

Second, unmeasured confounding variables could produce the observed covariation between GCs and survival. Our dynamic prediction analysis attempts to control for known predictors by holding major environmental influences on GCs constant and varying only the individual-specific random effects for simulated individuals with distinct GC histories. This analysis still identifies a robust association between high GCs and reduced survival. However, both elevated GCs and reduced survival may be caused independently by sustained differences in exposure to the unmeasured challenges faced or perceived by different individuals. Under this scenario, the individual differences in GC profiles that we observed could be viewed as the products of adaptive responses that allow individuals to cope with their different exposures (*6*, *21*). Hence, no deleterious effects of “chronic stress” would exist in this population beyond the costs imposed by the environmental stressors themselves. Although this interpretation is impossible to rule out, prior work in humans and other social primates has shown that exposure to chronic social stressors can lead to HPA axis dysfunction and stress-associated disease (*42*).

An important contribution of our study is to inform discussion of the prevailing evolutionary explanations for links between chronic stress, health, and fitness (*46*–*48*). These explanations recognize that the stress response evolved as an adaptive system to allow organisms to respond quickly and effectively to acute stressors (*1*). Specifically, activation of the stress response promotes fitness by supporting critical functions. On the other hand, in social systems in which organisms both need conspecifics for resource acquisition and defense and compete with conspecifics for limited resources (*1*), some aspects of social living have the potential to provoke repeated or sustained GC secretion, potentially leading to HPA axis dysregulation. In this context, an individual that experiences prolonged exposure to extreme disturbance (*40*) or sustained energetic or psychosocial stressors (*3*) can suffer damaged health and a shortened life span relative to individuals that do not have the same experience.

Our study is consistent with the hypothesis that shortened life spans in the face of adverse social conditions are mediated in part by differences in GC responses. Prior work with yellow baboons and mice indicates that social subordination may represent the root of one functional pathway from chronic psychosocial stress to poor health and increased mortality (*19*, *49*). However, the association between GC concentrations and survival in female baboons is not entirely attributable to covariation between GCs and social status: In our dynamic prediction analysis, the relationship persists after removing individual differences in social status. Hence, while low social status may play a role in mediating health and may even do so, in part, through GC-related mechanisms, additional proximate mechanisms must be invoked to explain life span disparities in female baboons. Other social gradients, including social integration and early-life adversity, are robustly linked to survival in both humans and nonhuman animals (*50*), including this baboon population (*37*, *38*). Recent work with the Amboseli baboons has shown that weak social bonds in adulthood do play a mediating role in pathways that may connect conditions experienced in early life with adult GC levels (*51*). A priority for future work should be to examine causal relationships between GCs and social gradients to understand the pathways through which they are linked to survival. Together, our findings indicate that GC levels are strong prognostic indicators of survival and may be key explanations of life span disparities in nonhuman primates and humans.

## MATERIALS AND METHODS

### Study system

Baboons (*Papio* spp., family Cercopithecidae) are large, predominantly terrestrial primates that inhabit a wide range of ecosystems and have a broad geographic distribution throughout most of Africa and part of the Arabian Peninsula. In the Amboseli ecosystem, baboons reside in relatively stable multimale-multifemale social groups of 18 to 130 individuals. Females in Amboseli reach menarche at a median age of 4.5 years and remain in their natal group throughout their lives (*52*). Both male and female baboons form linear dominance hierarchies. In females, dominance rank is maternally inherited in a pattern known as youngest ascendency: Adult daughters attain the rank immediately below that of their mother, in reverse order of age, although there is variation around this typical pattern (*53*).

The Amboseli basin in southern Kenya (2.667°S, 37.283°E) is a semiarid savannah mosaic that includes grasslands, shrubs, and scattered *Acacia*-dominated woodlands. The region experiences a dry season with virtually no rain from June to October and a wetter season from November to May (fig. S1). Total annual rainfall varies from 141 to 757 mm and averages about 350 mm (fig. S1) (*54*).

Baboons in the Amboseli basin have been studied intensively since 1971, with near-daily data collection. Data are collected from multiple study groups in which all animals are individually recognized by experienced observers. Collection of life history and behavioral data began in 1971, and noninvasive collection of fecal samples for steroid hormone analysis began in late 1999 (*22*). The population consists primarily of yellow baboons (*P. cynocephalus*), but there is a substantial level of admixture with anubis (olive) baboons (*P. anubis*) (*33*, *55*–*57*).

Our subjects are individually identified adult female baboons who reside throughout their lives in stable and highly cohesive social groups (that is, only male baboons disperse to new social groups). Observers census multiple social groups in the population on a near-daily basis to keep track of any changes in group membership, including disappearances, deaths, dispersals, and births. No female has ever permanently dispersed to a new group during the 49-year study, and our study population represents approximately half of the baboon population in the Amboseli ecosystem, suggesting that we would be very likely to detect even rare cases of permanent female movement between groups. Hence, whenever an adult female baboon disappears permanently from her social group, we assume that she has died. Deaths are seldom observed, and it is therefore rarely possible to identify causes of death. In addition, certain kinds of death are more likely to be observed than others, leading to major ascertainment bias for the subset of deaths for which we do assign causes. For example, deaths from predation are likely to occur at night, when no observers are present, whereas deaths associated with acute illness are more likely (but not certain) to be observed. Therefore, we analyzed all-cause mortality and are unable to break down mortality events into granular causes of death.

### Hormone sample collection and analysis

Fecal sample collection, storage, and extraction were carried out as described previously (*58*, *59*). Fecal samples were collected in the field by experienced observers during systematic behavioral data collection several days each week during 5-hour-long monitoring visits with baboon social groups. We used radioimmunoassays to measure fGC metabolites (*29*, *60*). Specifically, fGC concentrations were determined using the Corticosterone Double Antibody RIA Kit, designed for use in laboratory mice and rats (catalog no. 07120103; MP Biomedicals, Solon, OH, formerly ICN Diagnostics, Costa Mesa, CA; full laboratory protocols are available at https://amboselibaboons.nd.edu/). The primary antibody in this kit cross-reacts with major cortisol metabolites present in baboon feces (*61*). This RIA was validated for use with Amboseli baboon feces in (*59*), and the same assay has been used for analyzing GCs in Amboseli baboon fecal samples for nearly two decades. Interassay coefficients of variation were 13.6 and 10.7% (*n* = 49), respectively, for a low and high control. Intra-assay coefficients of variation were below 6% for both the low and high control (any duplicate above 15% was reassayed). All hormone values are expressed as nanogram per gram of dry feces. In this study, we used all fGC data for adult females living in 15 social groups from January 2000 through June 2019 (Fig. 1A). We excluded a few periods during which rates of data collection were low because of logistical challenges in the field (e.g., during political disruptions). We also excluded data collected during group fissions, which can take weeks to months, because we cannot measure dominance rank during these times, as group membership can change from one day to the next (i.e., the hierarchy membership changes). In the final dataset, the number of fGC measurements per female year ranged from 1 to 64, with a median of 7 (fig. S2).

### Covariates of fGC concentrations

Recent reviews have highlighted the challenges that complicate comparisons of endocrine traits of individuals across different times, locations, or environments (*5*, *14*, *62*). Specifically, if environmental stressors covary with both fitness and endocrine traits, then the fitness-reducing effects of environmental stressors can be falsely interpreted as a relationship between fitness and endocrine traits. To partially address this problem, our dynamic predictions hold constant the environmental challenges and intrinsic states that the individual was experiencing at the time of sample collection. Previous studies have described a variety of influences on fGC values of female baboons in our study population, and we drew on this knowledge to include additional predictors of fGC values in the longitudinal submodel (*28*–*32*). These influences, each of which is briefly summarized in the paragraphs that follow, were measured on the date of fecal sample collection or during a specific window preceding this date, as detailed below.

The age of the subject at the time of each fecal sample collection was based on subjects’ birthdates, which were known within an error of a few days for 228 of 242 subjects, based on our demographic records of pregnancies and births. Ages for the remaining 14 females were known within an error of 3 months due to short gaps in data collection.

Reproductive states included cycling, pregnant, or lactating, which were determined as described in (*29*), with one modification: We considered the first 4 days of lactation—which includes the day of parturition and the 3 days that follow—as “pregnant” to control for time lags in hormone secretion that make the first few days after parturition reflect the relatively high values of circulating steroid hormones during pregnancy (*30*).

Seasons included dry season (June to October) and wet season (November to May). These categories reflect the typical annual cycle of rainfall in the Amboseli basin, based on 43 years of daily weather data collected from the study site (fig. S1). In this study system, season is a proxy variable for predictable changes in nutritional status (apart from the influence of reproductive status). A previous study from this population found that nutritional state, assessed using thyroid hormone metabolites in feces, varies according to season (*63*). Specifically, female nutritional status is higher during the wet season, when green grass blades and fruits on which the baboons feed are abundant. Nutritional status is lower during the dry season, when baboons switch to foraging for underground grass corms that require more effort to obtain relative to their nutritional content.

Because there is interannual variation in the intensity and duration of the wet and dry seasons, we also calculated a 3-month rainfall anomaly as the difference between the total rainfall in the 3 months preceding sample collection and the long-term average for the same calendar dates based on the entire 43-year rainfall record. We followed the procedure used in (*51*) to account for within-season rainfall variability on fGC concentrations. We calculated the mean of daily maximum temperature in the 30 days before and including the day of sample collection because previous research has shown that, within seasons, fGC concentrations are elevated during months with relatively high daily maximum temperatures (*29*).

Group size was measured as the total number of adult animals in the focal female’s social group on the date of sample collection. We included group size squared in the model because previous research found a quadratic relationship between fGC and group size (*28*).

Prior research has shown that fGC values in this population are influenced by two distinct aspects of sample storage and processing (*31*). First, time to extraction is the elapsed time between the date of fecal sample collection and the date of methanol extraction. Second, time to assay is the elapsed time between the date of methanol extraction and the date on which the radioimmunoassay was completed.

We calculated monthly proportional dominance ranks for adult females based on all observed decided dyadic agonistic encounters between adult females. Proportional dominance rank, which ranges from 0 (lowest ranking) to 1 (highest ranking), expresses the proportion of adult females the focal female dominated. For each agonistic interaction, observers recorded the identities of the interactants and the outcome of the encounter. Decided interactions were those in which one individual displayed submissive signals and the other animal displayed either aggression or no agonistic signals. Undecided interactions did not contribute to the calculation of dominance ranks. The number of dyadic agonistic interactions in which both participants were adult females ranged from 85 to 1834 per month of observation, with a mean of 482.6 interactions used for constructing the adult female dominance hierarchy in each month.

Prior studies indicate that top-ranking individuals may differ qualitatively from other individuals in terms of how their fGC concentrations vary with social dominance (*31*, *64*). Therefore, we also included a binary indicator of top rank in the model, which equaled true only if the focal female outranked all other adult females in her social group on the date of sample collection.

We also evaluated the possibility that an individual’s genetic ancestry could influence its fGC values, although this variable has not been previously linked to fGC in baboons. As noted above, the Amboseli baboon population consists of individuals with varying degrees of admixture between yellow baboons (*P. cynocephalus*) and anubis baboons (*P. anubis*). An individual’s genetic hybrid score estimates the proportion of anubis ancestry in each individual’s genome [as described in (*33*)]. Hybrid scores were available for only 197 of 242 individuals in the current dataset, and hence, excluding all data from females without hybrid scores would reduce sample size considerably. To determine whether hybrid scores predict fGC, we fit a linear mixed model of log fGC that included hybrid score as a fixed effect, but that was otherwise specified identically to the longitudinal submodel from the main analysis (described in greater detail below). In this model, genetic hybrid score did not clearly predict fGC values (90% CI: [−0.105, 0.051]), and therefore, we excluded this variable from our main analysis.

### Time-varying covariates of adult female survival

We included three time-varying covariates of adult female survival: social bond strength measured using a dyadic sociality index with females (DSI_F_) and a dyadic sociality index with males (DSI_M_) and annual rainfall anomaly.

Dyadic bond strength with both male and female grooming partners strongly predicts survival in adult female baboons (*65*) [this measure is also highly correlated with the “social connectedness” metric that was linked to survival in (*38*)]. To quantify dyadic bond strength, we used grooming data to calculate age-specific indices of dyadic bond strength among all female-female and female-male dyads of adult animals that resided in the same group using methods modified from (*38*, *66*, *67*). The indices were calculated separately for each female in each adult year of her life, with a year of life corresponding to the period from one birthday to the next birthday, e.g., the seventh year of life lasts from the sixth birthday to the day before the seventh birthday. Years could be truncated on the right by the female’s death or by censoring at the end of observation.

Experienced observers collected systematic behavioral data several days each week during 5-hour-long contacts with baboon social groups. The observers recorded dyadic grooming during “representative interaction sampling,” in which observers moved through the group conducting 10-min-long focal animal samples (*68*), while recording all dyadic grooming interactions between any group members in their line of sight. The systematic nature of focal animal sampling is thus recapitulated in representative interaction sampling, as observers move from subject to subject in an order determined by a randomized list, ensuring representative sampling of the entire social group. Validation of the representative interaction sampling data and quantitative comparisons of sociality indices calculated from focal animal sampling and representative interaction sampling can be found in (*38*). Notably, the representative interaction sampling data provide a far richer and more complete record of female grooming interactions than the focal animal sampling data do.

Our grooming data consisted of counts of grooming events between adult females, with both the giver and receiver of grooming recorded. Grooming was recorded whenever one animal used both hands to pick through the fur of a second animal. Only grooming events between adult females, or between adult females and adult males, were considered for this analysis; grooming with juveniles was excluded. We excluded a few time periods during which rates of behavioral data collection were low because of logistical challenges in the field. Specifically, of the approximately 107 group years of monitoring on stable social groups since the beginning of the fGC data in January 2000, we excluded 17 brief periods of low observation intensity totaling 5.64 group years.

Estimating interaction rates from the representative interaction sampling data is complicated by the fact that the number of observers remained constant while group sizes varied. This resulted in higher numbers of grooming interactions being observed for any given dyad in a smaller group than in larger group. Following (*38*), we corrected for this issue by regressing daily grooming rates for each dyad against observer effort during the days that the dyad was observed. Considering each female year of life separately, we calculated daily grooming rates for each dyad in the population as the dyad’s number of grooming interactions (in either direction) divided by the number of co-residence days during that year. We calculated observer effort during the dyad’s co-residence days in a given group as the number of focal samples on adult females collected during those days, divided by the mean number of adult females in the group during those days, divided by the number of co-residence days. We then calculated an index of dyadic bond strength by taking the residuals of a regression between log daily grooming rate against log observer effort. Last, we calculated the two dyadic sociality indices that we included as covariates of survival for the focal female, bond strength with females (DSI_F_) and bond strength with males (DSI_M_), as the mean of the dyadic bond strength values for her three strongest adult female and adult male grooming partners, respectively. Rarely, some females had fewer than three total grooming partners of a given sex during a particular year (e.g., in unusually small groups with fewer than three adult males). In such cases, the indices were calculated as the mean bond strength values of however many partners of that sex the female had. In other words, we did not penalize the DSI_F_ or DSI_M_ values of females with fewer than three grooming partners of a given sex so as not to conflate bond quality with bond quantity. To summarize, higher DSI_F_ (or DSI_M_) values represent females with relatively strong bonds with top female (or male) grooming partners, given the distribution of normalized dyadic bond strength values among all adult female-female grooming pairs (or female-male grooming pairs) in the population during that year.

Annual rainfall anomaly is a measure of the degree to which the total rainfall during the focal individual life year deviated from the long-term average over the same calendar dates, based on the 43-year rainfall record. A previous study determined that adult female survival in this population is higher during years with higher rainfall (*69*). One possible mechanism for this effect is that key food resources, including grass corms on which baboons rely heavily during lean periods, may be more plentiful following periods of relatively abundant rain (*54*). To account for such rainfall-related influences on survival, we included annual rainfall anomaly as a covariate of adult female survival. We did not include social rank as a covariate of adult female survival because, in recent work, we have found no evidence that social rank predicts female survival in this population (*38*, *65*).

### Joint models of fGC and survival

Joint modeling of longitudinal and time-to-event data is a rapidly developing statistical approach that has been used in clinical studies to analyze the association between biomarkers that have been measured repeatedly and an event of interest, such as death (*27*, *70*–*74*). The approach involves simultaneously estimating two statistical models: (i) a longitudinal mixed-effects submodel that describes individual-specific change in the biomarker over time and (ii) a time-to-event submodel that analyzes the time until the event. The joint estimation is accomplished by specifying an association structure between the two submodels based on their shared parameters.

We fit joint models of longitudinally measured fGC and adult female survival under a Bayesian framework using the R package rstanarm version 2.19.3 (*34*, *35*). The submodel specifications and association structures are described in the paragraphs that follow. For each joint model, we used 8 Markov chains with 5000 total iterations each, including 2500 warm-up iterations, no thinning, and default (weakly informative) priors. We assessed model fit and chain convergence using Rhat, effective sample size, and Monte Carlo SE and by visually assessing trace plots for the model’s parameters (figs. S5 to S7 and tables S1 to S3).

#### Longitudinal submodel of fGC

In the joint models, we include specific aspects of individual-specific fGC trajectories as time-varying predictors of survival. To do so, we need to estimate the true, unobserved value of the underlying biomarker process *m_i_*(*t*) for individual *i* (*i* = 1,…, *N*) at time point *t*. We did so using a linear mixed-effects model in which the response variable was the natural log of fGC. Let *y_i_*(*t*) denote the value of the longitudinal outcome (natural log of fGC) for individual *i* at time point *t*. This individual’s complete record of observed fGC data consist of the measurements *y_ij_* = {*y_i_*(*t_ij_*), *j* = 1,…, *n_i_*} at time points *t_ij_*, where *n_i_* is the number of samples collected for individual *i*. We specify a linear mixed model for *y_i_* given a vector of random effects *b_i_*

$$\begin{array}{c} y_{i}(t)=m_{i}(t)+\varepsilon_{i}(t)=\\ X_{i}(t)\beta +Z_{i}(t)b_{i}+\varepsilon_{i}(t),\varepsilon_{i}(t)∼N(0,\sigma^{2}) \end{array}$$

where β is a vector of fixed effects with corresponding time-varying design matrix ***X****_i_*(*t*), ***b****_i_* is a vector of random effects with corresponding time-varying design matrix ***Z****_i_*(*t*), and ε*_i_*(*t*) is the error term with variance σ^2^. The fixed effects included all of the covariates of fGC described above and a fixed intercept, and the random effects included a random intercept of individual identity as well as a random slope of age for each individual. This random effects structure allowed each individual’s log fGC process to follow an individual-specific linear trajectory over the life course, after accounting for the fixed covariates. We *z*-score transformed the continuous covariates (3-month rainfall anomaly, maximum temperature, group size, group size squared, dominance rank, time to extraction, and time to assay) to make their estimates more directly comparable. The full longitudinal submodel explained about 21% of the variance in log fGC values (conditional *R*^2^ = 0.213), and the fixed effects alone explained around 14% of the variance in log fGC values (marginal *R*^2^ = 0.138).

#### Time-to-event submodel of adult female survival

The time-to-event submodel was a parametric proportional hazards model of adult female survival, with a flexible baseline hazard that we approximated using b-splines with two internal knots. Let $T_{i}=\text{min}(T_{i}^{*},C_{i})$ denote an observed event time (i.e., death), where $T_{i}^{*}$ is the potentially unobserved “true” event time for individual *i* and *C_i_* is the censoring time. We define an indicator function $\delta_{i}=I(T_{i}^{*}\leq C_{i})$, with *I*(·) taking the value of 1 when $T_{i}^{*}\leq C_{i}$ and 0 when otherwise. We model the hazard of death using a parametric proportional hazards model

$$h_{i}(t|M_{i}(t),w_{i}(t))=h_{0}(t)\text{exp}[\gamma^{T}w_{i}(t)+f\{\alpha ,M_{i}(t)\}]$$

where *M_i_*(*t*) = {*m_i_*(*s*), 0 ≤ *s* < *t*} is the history of the underlying longitudinal process (i.e., the true unobserved fGC trajectory) up to time *t*, *h_i_*(*t*) is the hazard of death for individual *i* at time *t*, *h*_0_(·) is the baseline hazard function, and ***w****_i_*(*t*) is a vector of exogenous and possibly time-varying covariates of survival with associated vectors of regression coefficients γ. The function *f*(·) represents the association structure that specifies which features or components of the longitudinal fGC process are included in the survival model; it is parameterized by a vector α (see the “Association structures” section below).

We included the exogenous time-varying covariates of adult female survival described above. The covariates were measured in 1-year time steps aligned to individual birthdays; for example, survival to the end of age class 7 was a function of DSI_F_, DSI_M_, and annual rain anomaly measured over the 1-year period extending from the animal’s sixth birthday to its seventh birthday. Covariate values for DSI_F_ were unavailable for 30 of 1634 (1.84%) of individual years, and covariate values for DSI_M_ values were unavailable for 52 of 1634 (3.18%) of individual years. Rather than discarding all of the fGC data that were collected during these individual years, we imputed the missing DSI_F_ and DSI_M_ values by setting them to the mean values for individuals of that age class. In addition, a subject-specific value of the longitudinal fGC process, as defined by the association structure (see below), was associated with the hazard of death at the same time point.

#### Association structures

We fit three different joint models in which we specified alternate plausible association structures between the longitudinal and time-to-event submodels and a fourth joint model with a null association structure (i.e., in which no aspect of the fGC trajectory was included as a predictor of survival). The current value association structure is given by

$$f\{\alpha ,M_{i}(t)\}=\alpha m_{i}(t)$$

the current slope association structure is given by

$$f\{\alpha ,M_{i}(t)\}=\alpha m_{i}^{\prime }(t),\text{with}\;m_{i}^{\prime }(t)=\frac{{\mathrm{dm}}_{i}(t)}{\mathrm{dt}}$$

and the cumulative effect or area under the curve association structure is given by

$$f\{\alpha ,M_{i}(t)\}=\alpha \int_{0}^{t}m_{i}(u)\mathrm{du}$$

## SUPPLEMENTARY MATERIALS

Supplementary material for this article is available at http://advances.sciencemag.org/cgi/content/full/7/17/eabf6759/DC1
